# Validation and refinement of the AJCC 8th edition ypStage in esophageal squamous cell carcinoma after neoadjuvant therapy: a large-scale retrospective cohort study

**DOI:** 10.1097/JS9.0000000000004776

**Published:** 2026-01-13

**Authors:** Zhiqiang Liu, Xiankai Chen, Xiufeng Wei, Jianjun Qin, Yong Li, Zhen Wang, Xiaobin Shang, Zhendong Huang, Fanmao Meng, Shao-Hua Xie, Jie He, Xiaozheng Kang, Ruixiang Zhang, Yin Li

**Affiliations:** a Section of Esophageal and Mediastinal Oncology, Department of Thoracic Surgery, National Cancer Center/National Clinical Research Center for Cancer/Cancer Hospital, Chinese Academy of Medical Sciences and Peking Union Medical College, Beijing, China; b Upper Gastrointestinal Surgery, Department of Molecular Medicine and Surgery, Karolinska Institutet, Karolinska University Hospital, Stockholm, Sweden

**Keywords:** cohort study, esophageal squamous cell carcinoma, neoadjuvant therapy, prognosis, recursive partitioning analysis, ypStage

## Abstract

**Background:**

The AJCC 8th edition post-neoadjuvant pathologic stage (ypStage) shows limited prognostic discrimination in esophageal squamous cell carcinoma (ESCC). Alternative strategies have been proposed, but most were limited by small samples and lack of inclusion of patients treated with immunotherapy. This study aimed to validate the AJCC 8th edition ypStage and develop a refined ypStage system with improved prognostic performance for ESCC.

**Methods:**

This was a large retrospective cohort study including 1848 consecutive ESCC patients treated with neoadjuvant therapy followed by esophagectomy between June 2018 and September 2023. Patients were randomly assigned to training (*n* = 1108) and validation (*n* = 740) cohorts. A refined ypStage was developed using recursive partitioning analysis (RPA). Survival outcomes were evaluated using Kaplan-Meier curves and log-rank tests, and multivariable Cox proportional hazards regression was used to confirm independent prognostic value. Prognostic performance of the RPA and AJCC 8th edition ypStage systems was compared using the Groome criteria: hazard consistency, hazard discrimination, sample balance, outcome prediction, and overall score. Subgroup analyses were performed by neoadjuvant therapy type.

**Results:**

The AJCC 8th edition ypStage showed a reversal of the expected survival hierarchy between ypStage II and IIIA [3-year overall survival (OS): II, 80.1%; IIIA, 90.7%]. RPA identified three groups: ypStage I (ypT0-2N0-1), II (ypT0-2N2-3 or ypT3-4bN0-1), and III (ypT3-4bN2-3). In the validation cohort, RPA ypStage demonstrated clear survival separation (3-year OS: I, 91.2%; II, 79.1%; III, 60.5%; pairwise *P* < 0.01). Multivariable analysis confirmed its independent prognostic value [II vs I: Hazard Ratio (HR) = 2.44, 95% Confidence Interval (CI): 1.68–3.52; III vs I: HR = 3.67, 95% CI: 2.28–5.90; both *P* < 0.001]. RPA ypStage outperformed AJCC ypStage across all evaluation metrics. Subgroup analyses further supported its robustness, particularly in patients receiving chemotherapy or chemo-immunotherapy.

**Conclusion:**

The RPA-derived ypStage provides improved prognostic stratification compared with the AJCC 8th edition in ESCC patients following neoadjuvant therapy. Further external validation is warranted for potential incorporation into future staging guidelines.

## Introduction

Esophageal cancer ranks 11th in global cancer incidence and seventh in cancer-related mortality[1]. Esophageal squamous cell carcinoma (ESCC), the predominant histologic subtype, carries a poor prognosis, with 5-year survival rates of less than 20% in patients with locally advanced disease.^[2–5]^ Neoadjuvant therapy, typically chemotherapy or chemoradiotherapy, followed by surgery, has become the standard of care, offering significant survival benefits over surgery alone.^[6–8]^ Recently, immunotherapy has been explored in combination with neoadjuvant regimens and shows promise in improving treatment response[9].

Accurate post-neoadjuvant staging is essential for guiding adjuvant therapy, risk stratification, and surveillance. Misclassification may lead to overtreatment or undertreatment, adversely affecting patient outcomes and healthcare resource utilization. The 8th edition of the American Joint Committee on Cancer (AJCC) introduced a dedicated post-neoadjuvant pathologic stage (ypStage) for esophageal cancer, without distinguishing between histologic subtypes[10]. Although some studies supported its prognostic validity, others reported unexpected survival inversions (i.e., a reversal of the expected survival hierarchy between ypStage II and IIIA), particularly in ESCC.^[11–16]^ However, these findings were based on relatively small cohorts.

Several alternative staging strategies have been explored, including tumor regression grade (TRG)-N staging, lymph node ratio-based classifications, and modified grouping schemes^[12,15,17]^. However, these studies were limited by small sample sizes and restricted to chemotherapy or chemoradiotherapy, without accounting for immunotherapy. The increasing use of neoadjuvant immunotherapy in ESCC highlights the urgent need to reassess and refine the existing staging system to reflect evolving clinical practice.

This study aimed to validate the AJCC 8th edition ypStage and to develop a refined ypStage system for ESCC. Using recursive partitioning analysis (RPA) in a large cohort treated with multiple neoadjuvant regimens, we sought to establish a data-driven ypStage system with improved prognostic stratification. We hypothesized that this refined system would provide superior prognostic discrimination compared with the AJCC 8th edition. A more accurate staging framework has the potential to guide individualized postoperative management, inform future staging guideline revisions, and reduce unnecessary treatment costs.

## Methods

### Study design and patient cohort

This large retrospective cohort study included 1848 consecutive patients with pathologically confirmed ESCC who underwent neoadjuvant therapy followed by esophagectomy between 1 June 2018 and 30 September 2023. Clinical and pathological data were retrieved from a prospectively maintained institutional database, with coding and variable definitions aligned to the international Esodata database standard, an international initiative aimed at standardizing esophagectomy data collection to ensure high-quality, comparable clinical data across centers worldwide[18]. Patients were excluded if they had distant metastases at diagnosis, incomplete clinical or pathological information, or were lost to follow-up at the first scheduled visit. The final cohort was randomly divided into a training set (*n* = 1108; 60%) and a validation set (*n* = 740; 40%). As this was a consecutive cohort including all eligible patients during the study period, no a priori sample size calculation was performed. Given the large sample size (*n* = 1848), the study had sufficient statistical power to detect clinically meaningful survival differences. This work has been reported in line with the STROCSS guidelines[19].

### Clinical and pathological variables

Clinical stage and ypStage were determined according to the 8th edition of the AJCC staging system. Clinical stage was determined before neoadjuvant therapy using endoscopic ultrasound (EUS), computed tomography (CT), positron emission tomography (PET)/CT, and endoscopic biopsy. All pathological evaluations were conducted by experienced gastrointestinal pathologists specializing in esophageal cancer. Residual tumor status was classified as R0 (no residual tumor), R1 (microscopic residual tumor), or R2 (macroscopic residual tumor), with all resection margins assessed according to the College of American Pathologists (CAP) guidelines for esophageal cancer[20]. TRG was assessed using the Mandard classification[21], which consists of five grades: TRG1, no residual tumor with fibrosis extending through all layers of the esophageal wall; TRG2, rare residual tumor cells scattered within dense fibrosis; TRG3, increased number of residual tumor cells, but fibrosis remains predominant; TRG4, residual tumor outgrowing the fibrotic component; and TRG5, no histological evidence of tumor regression with extensive residual tumor. Perineural invasion and vascular invasion were recorded as either present or absent based on microscopic examination. For each patient, the total number of dissected lymph nodes and the number of metastatic (positive) lymph nodes were recorded, and the lymph node ratio was calculated as the proportion of positive to total dissected nodes. Additional variables collected included age, sex, body mass index (BMI), smoking status, alcohol drinking status, type of neoadjuvant therapy (chemotherapy alone, radiotherapy alone, chemoradiotherapy, chemo-immunotherapy, or chemo-radio-immunotherapy), surgical approach, tumor location, ypT and ypN categories, tumor differentiation grade, and receipt of postoperative adjuvant therapy.

**HIGHLIGHTS**

- The AJCC 8th edition ypStage showed inconsistent prognostic hierarchy in esophageal squamous cell carcinoma (ESCC), with survival inversion between stages II and IIIA.
- A refined ypStage system was established using recursive partitioning analysis (RPA).
- The RPA ypStage demonstrated superior prognostic performance over the AJCC 8th edition ypStage system across multiple evaluation criteria.
- This study presents the first large-scale ESCC ypStage system incorporating patients treated with immunotherapy, with potential to guide individualized postoperative strategies and inform future staging guideline revisions.

### Follow-up and overall survival

Postoperative surveillance was conducted at regular intervals in accordance with institutional guidelines: every 3 months during the first 2 years after surgery, every 6 months for the following 3 years, and annually thereafter. Survival status was determined through outpatient visits, telephone follow-up, and review of medical records. Follow-up continued until death or the end of the study period (30 September 2024), whichever occurred first.

The primary endpoint was overall survival (OS), defined as the time from surgery to death from any cause or the date of last contact. Patients who were alive at the last follow-up were censored.

### Statistical analysis

Patient characteristics were summarized as medians with interquartile ranges (IQRs) for continuous variables and as frequencies with percentages for categorical variables. Variables were compared between cohorts using the Mann–Whitney *U* test for continuous variables and χ^2^ tests (or Fisher’s exact tests when appropriate) for categorical variables. Kaplan-Meier survival curves were generated and compared using the log-rank test; pairwise *P*-values were adjusted for multiple comparisons using the Benjamini–Hochberg method.

RPA was conducted to construct a refined staging system based on ypT and ypN categories, the core components of the AJCC system, to ensure clinical relevance and enhance clinical applicability[22]. The algorithm identified homogeneous survival groups by iteratively splitting the dataset using log-rank tests with a significance threshold of *P* < 0.05. Partitioning was terminated when no further statistically significant splits could be made. Terminal nodes were subsequently evaluated for 3-year OS and aggregated into three broader groups – RPA ypStage I, II, and III – based on survival similarity and clinical interpretability. The RPA model was developed using a validated online platform (http://rpa.renlab.org/)[23].

To assess the independent prognostic value of the AJCC 8th edition ypStage and the new RPA ypStage, both univariable and multivariable Cox proportional hazards regression analyses were conducted. Variables with *P* < 0.05 in univariable analysis were entered into the multivariable model, followed by backward stepwise selection with a retention threshold of *P* < 0.05.

The prognostic performance of the new RPA ypStage and the AJCC 8th edition ypStage was evaluated using the well-accepted Groome criteria,^[24–26]^ which comprise five metrics: hazard consistency (similarity of survival rates within stage groups), hazard discrimination (differences in survival across stage groups), sample size balance (distribution uniformity across stages), outcome prediction (proportion of survival variation explained), and an overall score summarizing the four individual metrics. Each metric was assessed in both the training and validation cohorts. The two staging systems were ranked based on normalized scores, with lower scores indicating better performance. Overall staging performance was compared using 2000 bootstrap replications. To enhance interpretability, radar plots were generated to visually compare the two systems across the five metrics.

Subgroup analyses by neoadjuvant therapy were pre-specified as exploratory and included chemotherapy-alone, chemo-immunotherapy, chemoradiotherapy, and chemo-radio-immunotherapy; the radiotherapy-alone subgroup was excluded owing to insufficient size. Survival across RPA ypStage categories was estimated with Kaplan-Meier curves and compared using pairwise log-rank tests.

All statistical analyses were performed using R software (version 4.5.0; R Foundation for Statistical Computing, Vienna, Austria), and all *P*-values were two-sided.

## Results

### Patient characteristics

Patient characteristics of the whole (*n* = 1848), training (*n* = 1108), and validation (*n* = 740) cohorts are summarized in Table 1. The median follow-up duration was 23 months (interquartile range: 12 to 35 months; maximum: 64 months). The median age was 62.0 years (IQR, 57.0–67.0), and 1583 (85.7%) were male. Neoadjuvant therapy included chemotherapy alone (*n* = 707, 38.3%), radiotherapy alone (*n* = 11, 0.6%), chemoradiotherapy (*n* = 225, 12.2%), chemo-immunotherapy (*n* = 792, 42.9%), and chemo-radio-immunotherapy (*n* = 113, 6.1%). Minimally invasive esophagectomy was performed in 1714 (92.7%) patients, with R0 resection achieved in 1755 (95.0%). The ypStage distribution according to the AJCC 8th edition was: Stage I, 823 (44.5%); Stage II, 224 (12.1%); Stage IIIA, 231 (12.5%); Stage IIIB, 453 (24.5%); and Stage IVA, 117 (6.3%). A complete pathological response (TRG1) was observed in 540 patients (29.2%). Pre-treatment PET/CT was performed in 1631 patients (88.3%) to assist clinical staging, in addition to standard modalities such as EUS, CT, and endoscopic biopsy. Other clinicopathological variables, including BMI, smoking and alcohol drinking status, clinical stage, tumor location, ypT and ypN categories, tumor differentiation, perineural invasion, vascular invasion, the numbers of dissected and positive lymph nodes, the lymph node ratio, and receipt of postoperative adjuvant therapy are detailed in Table 1. Baseline characteristics between the training and validation cohorts were broadly comparable, with *P* values for each variable reported in Table 1.

**Table 1:** Patient characteristics.

Characteristics	Whole cohort (*n* = 1848)	Training cohort (*n* = 1108)	Validation cohort (*n* = 740)	*P*
Age, years	62.0 (57.0, 67.0)	62.0 (57.0, 67.0)	62.0 (56.0, 67.0)	0.620
< 65	1141 (61.7)	676 (61.0)	465 (62.8)	0.457
≥ 65	707 (38.3)	432 (39.0)	275 (37.2)	
Sex				0.647
Female	265 (14.3)	155 (14.0)	110 (14.9)	
Male	1583 (85.7)	953 (86.0)	630 (85.1)	
BMI, kg/m^2^	23.2 (21.2, 25.2)	23.2 (21.2, 25.3)	23.3 (21.3, 25.2)	0.826
Smoking status				0.583
Never	647 (35.0)	384 (34.7)	263 (35.5)	
Former	968 (52.4)	590 (53.2)	378 (51.1)	
Current	233 (12.6)	134 (12.1)	99 (13.4)	
Alcohol drinking status				0.570
Never	577 (31.2)	342 (30.9)	235 (31.8)	
Occasional	213 (11.5)	127 (11.5)	86 (11.6)	
Former	403 (21.8)	233 (21.0)	170 (23.0)	
Current	655 (35.4)	406 (36.6)	249 (33.6)	
Clinical stage (AJCC 8th edition)				0.328
I	505 (27.3)	298 (26.9)	207 (28.0)	
II	443 (24.0)	262 (23.6)	181 (24.5)	
III	802 (43.4)	496 (44.8)	306 (41.4)	
IVA	98 (5.3)	52 (4.7)	46 (6.2)	
Neoadjuvant therapy				0.650
Chemotherapy alone	707 (38.3)	439 (39.6)	268 (36.2)	
Radiotherapy alone	11 (0.6)	7 (0.6)	4 (0.5)	
Chemoradiotherapy	225 (12.2)	130 (11.7)	95 (12.8)	
Chemo-immunotherapy	792 (42.9)	467 (42.1)	325 (43.9)	
Chemo-radio-immunotherapy	113 (6.1)	65 (5.9)	48 (6.5)	
Surgical approach				0.078
MIE	1714 (92.7)	1023 (92.3)	691 (93.4)	
RAMIE	118 (6.4)	71 (6.4)	47 (6.4)	
Open	16 (0.9)	14 (1.3)	2 (0.3)	
Residual tumor status				0.250
R0	1755 (95.0)	1046 (94.4)	709 (95.8)	
R1	69 (3.7)	44 (4.0)	25 (3.4)	
R2	24 (1.3)	18 (1.6)	6 (0.8)	
Tumor location				0.716
Cervical	22 (1.2)	11 (1.0)	11 (1.5)	
Upper thoracic	274 (14.8)	168 (15.2)	106 (14.3)	
Middle thoracic	734 (39.7)	444 (40.1)	290 (39.2)	
Lower thoracic	818 (44.3)	485 (43.8)	333 (45.0)	
ypT (AJCC 8th edition)				0.464
T0	405 (21.9)	242 (21.8)	163 (22.0)	
Tis	123 (6.7)	66 (6.0)	57 (7.7)	
T1	391 (21.2)	245 (22.1)	146 (19.7)	
T2	271 (14.7)	159 (14.4)	112 (15.1)	
T3	618 (33.4)	376 (33.9)	242 (32.7)	
T4a	37 (2.0)	19 (1.7)	18 (2.4)	
T4b	3 (0.2)	1 (0.1)	2 (0.3)	
ypN (AJCC 8th edition)				0.032
N0	1063 (57.5)	622 (56.1)	441 (59.6)	
N1	454 (24.6)	294 (26.5)	160 (21.6)	
N2	238 (12.9)	145 (13.1)	93 (12.6)	
N3	93 (5.0)	47 (4.2)	46 (6.2)	
ypStage (AJCC 8th edition)				0.183
I	823 (44.5)	488 (44.0)	335 (45.3)	
II	224 (12.1)	129 (11.6)	95 (12.8)	
IIIA	231 (12.5)	151 (13.6)	80 (10.8)	
IIIB	453 (24.5)	278 (25.1)	175 (23.6)	
IVA	117 (6.3)	62 (5.6)	55 (7.4)	
Tumor differentiation				0.091
Well differentiated (G1)	39 (2.1)	17 (1.5)	22 (3.0)	
Moderately differentiated (G2)	618 (33.4)	387 (34.9)	231 (31.2)	
Poorly differentiated (G3)	534 (28.9)	318 (28.7)	216 (29.2)	
Undifferentiated (G4)	18 (1.0)	13 (1.2)	5 (0.7)	
Uncertain (Gx)	639 (34.6)	373 (33.7)	266 (35.9)	
Tumor regression grade (TRG)				0.941
TRG1	540 (29.2)	315 (28.4)	225 (30.4)	
TRG2	274 (14.8)	164 (14.8)	110 (14.9)	
TRG3	401 (21.7)	244 (22.0)	157 (21.2)	
TRG4	537 (29.1)	325 (29.3)	212 (28.6)	
TRG5	56 (3.0)	36 (3.2)	20 (2.7)	
Unknown	40 (2.2)	24 (2.2)	16 (2.2)	
Perineural invasion				0.570
No	1447 (78.3)	873 (78.8)	574 (77.6)	
Yes	401 (21.7)	235 (21.2)	166 (22.4)	
Vascular invasion				0.746
No	1508 (81.6)	901 (81.3)	607 (82.0)	
Yes	340 (18.4)	207 (18.7)	133 (18.0)	
Number of lymph nodes dissected	35.0 (28.0, 45.2)	35.0 (27.8, 45.0)	36.0 (28.8, 46.0)	0.152
Number of positive lymph nodes	0.00 (0.00, 2.00)	0.00 (0.00, 2.00)	0.00 (0.00, 2.00)	0.485
Lymph node ratio	0.00 (0.00, 0.05)	0.00 (0.00, 0.05)	0.00 (0.00, 0.05)	0.427
Postoperative adjuvant therapy				0.697
No	1135 (61.4)	685 (61.8)	450 (60.8)	
Yes	713 (38.6)	423 (38.2)	290 (39.2)	

AJCC, American Joint Committee on Cancer; BMI, body mass index; MIE, minimally invasive esophagectomy; RAMIE, robot-assisted minimally invasive esophagectomy.

Data are median (interquartile range) or number (%). Two-sided *P* values compare the Training and Validation cohorts. “Radiotherapy alone” refers to preoperative radiation without concurrent systemic therapy owing to chemotherapy contraindications, intolerance, or multidisciplinary team decisions.

Tumor regression grade (TRG) was assessed in five tiers according to the Mandard Classification: TRG1, complete response with no residual tumor; TRG2, rare scattered tumor cells within fibrosis; TRG3, fibrosis predominant over residual tumor; TRG4, residual tumor predominant over fibrosis; and TRG5, no regression with extensive residual tumor.

### Validation of AJCC 8th edition ypStage

**Figure 1.**
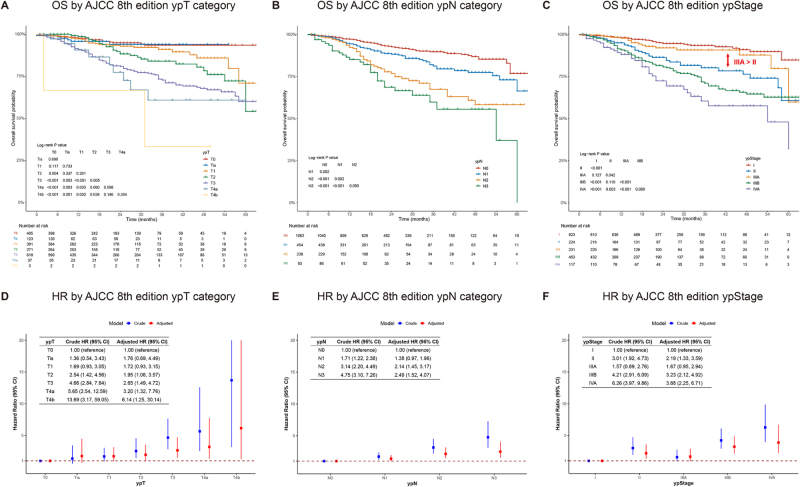
Prognostic validation of the AJCC 8th edition ypStage in the whole cohort. (A–C) Kaplan–Meier survival curves stratified by AJCC 8th edition ypT category (A), ypN category (B), and ypStage (C). All pairwise log-rank *P*-values are shown in the respective panels. In panel C, the reversal of the expected survival hierarchy between ypStage II and IIIA is indicated by a red arrow (ypStage IIIA > II). (D–F) Multivariable Cox proportional hazards models estimating hazard ratios (HRs) for overall survival (OS) by ypT category (D), ypN category (E), and ypStage (F). Blue bars indicate crude HRs with 95% confidence intervals; red bars indicate adjusted HRs with 95% confidence intervals. Covariates for adjustment included: age, BMI, smoking status, neoadjuvant therapy, residual tumor status, perineural invasion, vascular invasion, and ypN category (for ypT category); age, BMI, smoking status, neoadjuvant therapy, residual tumor status, perineural invasion, vascular invasion, and ypT category (for ypN category); age, BMI, smoking status, neoadjuvant therapy, residual tumor status, perineural invasion, and vascular invasion (for ypStage).

In the whole cohort (*n* = 1848), the ypT and ypN categories were associated with a monotonic decline in OS (Fig. 1A and D for ypT; 1B and E for ypN). In contrast, the AJCC 8th edition ypStage failed to maintain a hierarchical survival pattern. Specifically, patients with ypStage IIIA had better OS than those with ypStage II (Fig. 1C). Three-year OS rates were 93.3% for ypStage I, 80.1% for ypStage II, 90.7% for ypStage IIIA, 70.9% for ypStage IIIB, and 60.4% for ypStage IVA (Supplemental Digital Content Table 1A, available at: http://links.lww.com/JS9/G650). In multivariable analysis (adjusted for age, BMI, smoking status, neoadjuvant therapy, residual tumor status, perineural invasion, and vascular invasion), hazard ratios (HRs) did not show a consistent increase across stages: ypStage II (HR = 2.19), IIIB (HR = 3.23), and IVA (HR = 3.88) were independently associated with worse survival compared to ypStage I, whereas ypStage IIIA was not [HR = 1.67; 95% confidence interval (CI): 0.95–2.94; *P* = 0.077; Fig. 1F and Supplemental Digital Content Table 2, available at: http://links.lww.com/JS9/G650]. These findings suggested that the current AJCC 8th edition ypStage system lacked hierarchical prognostic order and might require refinement to improve risk stratification in patients with ESCC after neoadjuvant therapy.

### Development of the RPA ypStage

**Figure 2.**
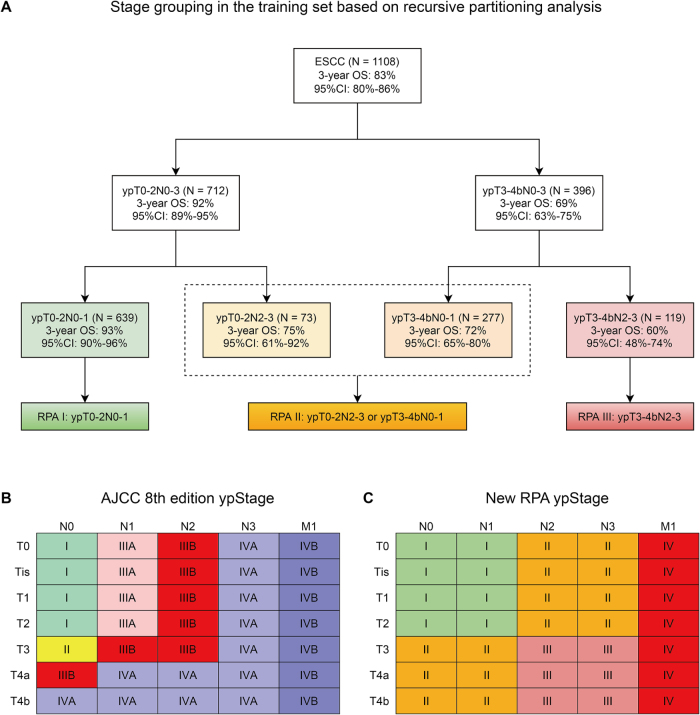
Development of the RPA ypStage in the training cohort. (A) Recursive partitioning analysis (RPA) tree based on ypT and ypN categories in the training cohort. (B and C) Comparison of stage groupings between the AJCC 8th edition ypStage (B) and the newly developed RPA ypStage (C).

In the training cohort (*n* = 1108), RPA was performed using ypT and ypN categories to identify distinct prognostic groups (Fig. 2A). The initial split was based on ypT, dividing patients into ypT0-2N0-3 and ypT3-4bN0-3 groups according to 3-year OS. Further stratification by nodal status (N0-1 vs N2-3) resulted in four terminal nodes. These nodes were subsequently consolidated into three prognostic stages based on survival similarity and clinical interpretability. The final RPA ypStage system defined ypStage I as ypT0-2N0-1, ypStage II as ypT0-2N2-3 or ypT3-4bN0-1, and ypStage III as ypT3-4bN2-3. Stage distributions based on the AJCC 8th edition ypStage and the new RPA ypStage are shown in Figure 2B and C, respectively.

### Validation of the RPA ypStage

**Figure 3.**
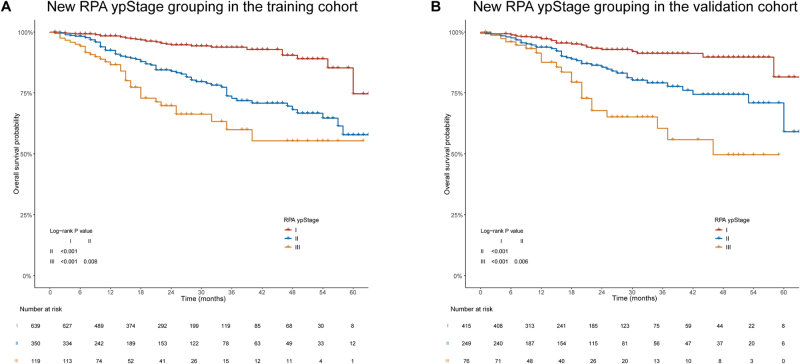
Prognostic performance of the RPA ypStage in the training and validation cohorts. (A and B) Kaplan–Meier survival curves stratified by RPA ypStage I-III in the training cohort (A) and validation cohort (B).

The RPA ypStage was further validated in both the training and validation cohorts and demonstrated strong prognostic discrimination. Kaplan-Meier survival curves showed clear separation among RPA ypStages I-III in both the training (Fig. 3A) and validation (Fig. 3B) cohorts, with statistically significant pairwise differences between stages (all log-rank *P* < 0.01). In the training cohort, 3-year OS rates were 93.7% for ypStage I, 72.8% for ypStage II, and 59.9% for ypStage III; in the validation cohort, 3-year OS rates were 91.2%, 79.1%, and 60.5%, respectively (Supplemental Digital Content Table 1B, available at: http://links.lww.com/JS9/G650).

Subgroup analyses stratified by neoadjuvant therapy type further supported the robustness of the RPA ypStage. Among patients who received chemotherapy-alone, chemo-immunotherapy, chemoradiotherapy, or chemo-radio-immunotherapy, the RPA ypStage consistently differentiated survival across the three stage groups (Supplemental Digital Content Figure 1A–D, available at: http://links.lww.com/JS9/G650). The overall trends remained consistent, though separation was less distinct in the chemoradiotherapy and chemo-radio-immunotherapy subgroups, likely due to limited sample sizes in ypStage III.

Multivariable Cox regression (adjusted for age, BMI, smoking status, neoadjuvant therapy, residual tumor status, perineural invasion, and vascular invasion) confirmed the independent prognostic value of the RPA ypStage. Compared with ypStage I, the adjusted hazard ratio was 2.44 (95% CI: 1.68–3.52; *P* < 0.001) for ypStage II and 3.67 (95% CI: 2.28–5.90; *P* < 0.001) for ypStage III (Supplemental Digital Content Table 3, available at: http://links.lww.com/JS9/G650).

### Performance comparison between ypStage systems

**Figure 4.**
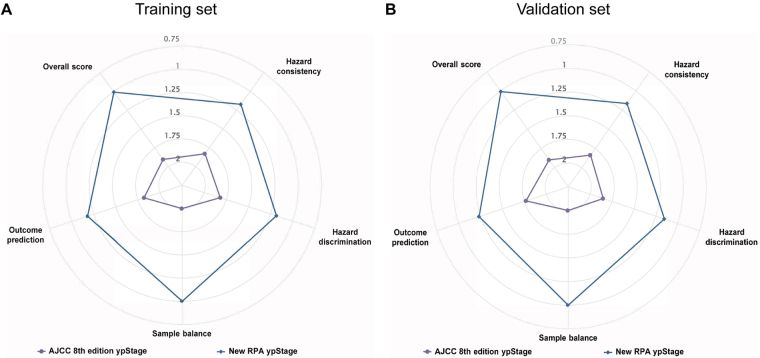
Comparative performance of the RPA ypStage and the AJCC 8th edition ypStage. (A and B) Radar plots illustrating staging performance across five Groome criteria (hazard consistency, hazard discrimination, sample balance, outcome prediction, and overall score) in the training cohort (A) and validation cohort (B). Lower scores indicate better performance. A larger polygon area reflects better overall performance.

Comparative analysis using the Groome criteria showed that the RPA ypStage outperformed the AJCC 8th edition ypStage across all five domains. It demonstrated better hazard consistency, greater hazard discrimination, more balanced stage distribution, and improved outcome prediction. In 2000 bootstrap replications, the RPA ypStage was ranked first in over 90% of iterations in both the training and validation cohorts (Fig. 4; Supplemental Digital Content Table 4, available at: http://links.lww.com/JS9/G650).

## Discussion

In this study, we developed and validated a refined RPA ypStage for patients with ESCC undergoing neoadjuvant therapy followed by esophagectomy. Compared with the current AJCC 8th edition ypStage system, which showed inconsistent prognostic hierarchy, the new RPA ypStage system provided superior prognostic stratification. It demonstrated clear separation of survival curves between stage groups, robust performance in both training and validation cohorts, and consistent performance across treatment subgroups, especially in chemotherapy-alone and chemo-immunotherapy subgroups. These findings highlight the limitations of the 8th edition ypStage and support the potential of a data-driven recursive partitioning approach to improve the accuracy of post-neoadjuvant pathologic staging in ESCC.

Although the AJCC 8th edition ypStage system was developed using large international multicenter datasets and well-established staging principles, it applies a single ypStage classification across all histological subtypes, without distinguishing between squamous cell carcinoma and adenocarcinoma[10]. While a retrospective cohort study demonstrated better predictive performance of the AJCC 8th edition ypTNM stage grouping compared with the 7th edition, its survival analyses were simplified into four merged stages due to small sample sizes and were restricted to patients who received chemotherapy[11]. However, several other studies have reported prognostic inconsistencies within the current AJCC 8th edition ypStage system, including survival inversion between ypStage II and IIIA.^[12–16]^ A similar pattern was also observed in our cohort and further confirmed by multivariable analysis with larger sample sizes, underscoring the limited prognostic utility of the current AJCC 8th edition ypStage system.

Several alternative approaches have been proposed to improve the post-neoadjuvant prognostic stratification in esophageal cancer. Wong *et al*[15] introduced a TRG-N system integrating TRG with the ypN category in esophageal squamous cell carcinoma, reporting superior prognostic discrimination over the AJCC 8th edition; however, the study was limited by the small sample size, insufficient survival separation between certain stages, and the inherent subjectivity of TRG assessment. Zhang *et al*[17] proposed a revised ypN classification based on lymph node ratio, which improved prognostic performance in a predominantly adenocarcinoma cohort. This approach simply replaced the ypN category with lymph node ratio without re-evaluating the overall staging structure, and its applicability remains uncertain in patients receiving multimodal neoadjuvant regimens beyond chemoradiotherapy. Oshikiri *et al*[14] proposed a modified ypStage system by unifying the AJCC 8th edition and the Japanese classification for patients with ESCC treated with neoadjuvant chemotherapy. Their model improved prognostic discrimination by addressing survival inversion between ypStage II and IIIA, but was limited to chemotherapy-treated patients and did not account for those receiving chemoradiotherapy or immunotherapy. Additionally, Park et al[12]. proposed a new ypStage grouping system for patients with ESCC treated with neoadjuvant chemoradiotherapy, also using recursive partitioning to revise the AJCC classification. While their system addressed the survival inversion between ypStage II and IIIA and improved prognostic discrimination, the study was limited to patients treated with chemoradiotherapy and lacked multivariable adjustment for potential confounders.

In this study, we developed the RPA ypStage using a recursive partitioning algorithm that grouped patients with similar survival outcomes. This data-driven approach allows the stage structure to directly reflect survival patterns while retaining the conventional ypT and ypN parameters of the current AJCC system. Specifically, Stage II combines ypT0-2N2-3 and ypT3-4bN0-1, two intermediate-risk post-neoadjuvant phenotypes with comparable hazards. The ypT0-2N2-3 phenotype indicates limited mural invasion with substantial residual nodal metastasis, suggesting treatment resistance and a higher risk of systemic dissemination. The ypT3-4bN0-1 phenotype represents deep local invasion with absent or minimal nodal involvement, indicating predominantly locoregional aggressiveness. In both the training and validation cohorts, these patterns showed similar adjusted hazard ratios and survival intermediate between those of Stage I and Stage III, supporting their placement in a single prognostic stratum by the RPA algorithm. This interpretation is pragmatic and hypothesis-generating and will be examined in planned multicenter external validation.

The RPA ypStage was derived from a large ESCC cohort treated with various neoadjuvant regimens, including chemotherapy alone, chemo-immunotherapy, chemoradiotherapy, and chemo-radio-immunotherapy, which may enhance its applicability across different clinical scenarios. Its independent prognostic value was confirmed by multivariable analysis, and superior performance was demonstrated across established evaluation metrics, supporting its potential generalizability and clinical utility in guiding postoperative management. In addition, the model was developed and internally validated using a large, prospectively maintained database from a high-volume esophageal cancer center, further supporting its robustness and clinical relevance. Measures such as consecutive case inclusion, standardized data definitions aligned with Esodata, and multidisciplinary review of staging and pathology were undertaken to minimize selection and information bias.

Although relying solely on ypT and ypN categories may not fully capture the biological complexity and treatment response in ESCC, this approach offers the advantage of simplicity and facilitates clinical implementation. Future staging refinements may consider incorporating additional indicators, such as treatment response or biological markers, potentially through machine learning algorithms. However, such efforts may increase model complexity while reducing interpretability and clinical applicability. The RPA ypStage developed in our study retains the use of conventional pathological parameters while enhancing prognostic stratification, striking a balance between simplicity and performance. Its straightforward structure and reliable prognostic value make it well suited for real-world clinical applications, including treatment planning and trial stratification.

The RPA ypStage system has potential to guide postoperative management. Patients classified as RPA ypStage III had poorer survival and may warrant consideration of more intensive surveillance; whether escalation of adjuvant therapy guided by RPA ypStage improves outcomes requires prospective evaluation. Conversely, the favorable prognosis in RPA ypStage I suggests that de-escalation of adjuvant therapy or follow-up intensity could be appropriate in selected cases, but this also requires validation. These prognostic differences support the use of the RPA ypStage for risk-adapted, individualized postoperative strategies in clinical practice. However, treatment decisions should continue to follow current guidelines and multidisciplinary team review, as this study did not evaluate the effects of adjuvant therapies. Any consideration of treatment escalation or de-escalation based on the RPA ypStage should be regarded as hypothesis-generating rather than a treatment recommendation.

To facilitate clinical adoption, we will initiate external validation through a multicenter collaboration network, which is developing a nationwide esophageal cancer database aligned with internationally recognized data definitions and standardized follow-up protocols. In collaboration with both domestic and international centers, we will validate the RPA ypStage across diverse clinical settings and neoadjuvant regimens. We also plan to integrate data from ongoing prospective studies and clinical trials to further assess the clinical utility and generalizability of the system.

This study has several limitations. First, the study was limited to patients with ESCC, and the findings may not be generalizable to esophageal adenocarcinoma. Second, although the RPA ypStage system has been internally validated, external validation in multicenter cohorts with diverse treatment regimens and surgical approaches is warranted to assess its generalizability. Third, certain treatment subgroups, particularly those receiving chemoradiotherapy or chemo-radio-immunotherapy, had relatively small sample sizes, which may have limited the power to detect prognostic differences. Interpretation of these subgroup analyses should therefore be approached with caution. Fourth, the follow-up period was relatively short in some patients, and longer-term data are needed to assess the durability of survival stratification; we will update the analyses when longer follow-up data become available. Finally, the refined RPA ypStage is based solely on ypT and ypN and therefore does not capture dynamic treatment response. TRG was excluded to maintain simplicity and cross-center generalizability, given heterogeneity in grading and reporting as well as interobserver variability. In planned multicenter external validation, we will assess whether incorporating a harmonized TRG or other response-based indicators provides incremental prognostic value.

In conclusion, the RPA ypStage provides a refined post-neoadjuvant pathologic staging system for patients with ESCC, with the potential to improve postoperative prognostic assessment and support individualized care. This study proposes the first ESCC ypStage system derived from a large cohort that includes patients treated with neoadjuvant immunotherapy. Further multicenter validation is warranted, along with consideration for its integration into future international staging frameworks.

## Data Availability

Data are available from the authors upon reasonable request.

## Research registration unique identifying number (UIN)

Name of the registry: Research Registry. Unique identifying number or registration ID: researchregistry11561. Hyperlink to your specific registration (must be publicly accessible and will be checked): https://www.researchregistry.com/browse-the-registry/#home/registrationdetails/68d6b2227b250302c0720a1d/.

## References

[1] Bray F Laversanne M Sung H. Global cancer statistics 2022: GLOBOCAN estimates of incidence and mortality worldwide for 36 cancers in 185 countries. CA Cancer J Clin 2024;74:229–63. 38572751 10.3322/caac.21834

[2] Anderson LA Tavilla A Brenner H. Survival for oesophageal, stomach and small intestine cancers in Europe 1999-2007: results from EUROCARE-5. Eur J Cancer 2015;51:2144–57. 26421818 10.1016/j.ejca.2015.07.026 PMC5729902

[3] Zeng H Chen W Zheng R. Changing cancer survival in China during 2003–15: a pooled analysis of 17 population-based cancer registries. Lancet Glob Health 2018;6:e555–e567. 29653628 10.1016/S2214-109X(18)30127-X

[4] Pennathur A Gibson MK Jobe BA Luketich JD. Oesophageal carcinoma. Lancet 2013;381:400–12. 23374478 10.1016/S0140-6736(12)60643-6

[5] Abnet CC Arnold M Wei W-Q. Epidemiology of esophageal squamous cell carcinoma. Gastroenterology 2018;154:360–73. 28823862 10.1053/j.gastro.2017.08.023 PMC5836473

[6] van Hagen P Hulshof MC van Lanschot JJ. Preoperative chemoradiotherapy for esophageal or junctional cancer. N Engl J Med 2012;366:2074–84. 22646630 10.1056/NEJMoa1112088

[7] Yang H Liu H Chen Y. Long-term efficacy of neoadjuvant chemoradiotherapy plus surgery for the treatment of locally advanced esophageal squamous cell carcinoma: the NEOCRTEC5010 randomized clinical trial. JAMA Surg 2021;156:721–29. 34160577 10.1001/jamasurg.2021.2373 PMC8223138

[8] Kato K Machida R Ito Y. Doublet chemotherapy, triplet chemotherapy, or doublet chemotherapy combined with radiotherapy as neoadjuvant treatment for locally advanced oesophageal cancer (JCOG1109 NExT): a randomised, controlled, open-label, phase 3 trial. Lancet 2024;404:55–66. 38876133 10.1016/S0140-6736(24)00745-1

[9] Qin J Xue L Hao A. Neoadjuvant chemotherapy with or without camrelizumab in resectable esophageal squamous cell carcinoma: the randomized phase 3 ESCORT-NEO/NCCES01 trial. Nat Med 2024;30:2549–57. 38956195 10.1038/s41591-024-03064-w PMC11405280

[10] Rice TW Ishwaran H Ferguson MK Blackstone EH Goldstraw P. Cancer of the esophagus and esophagogastric junction: an eighth edition staging primer. J Thorac Oncol 2017;12:36–42. 27810391 10.1016/j.jtho.2016.10.016 PMC5591443

[11] Sudo N Ichikawa H Muneoka Y. Clinical utility of ypTNM stage grouping in the 8th edition of the American Joint Committee on Cancer TNM staging system for esophageal squamous cell carcinoma. Ann Surg Oncol 2021;28:650–60. 33025354 10.1245/s10434-020-09181-3

[12] Park SY Park B Yun JK. proposal of new ypStage grouping system for esophageal squamous cell carcinoma patients who underwent neoadjuvant chemoradiotherapy followed by surgery. Ann Surg 2025;281:288–95. 38230528 10.1097/SLA.0000000000006204

[13] Yuan C Wu X Yang Y. Clinical characteristics and survival of esophageal cancer patients: annual report of the surgical treatment in Shanghai Chest Hospital, 2017. J Thorac Dis 2024;16:2948–62. 38883642 10.21037/jtd-24-49 PMC11170405

[14] Oshikiri T Goto H Kato T. Proposed modification of the eighth edition of the AJCC-ypTNM staging system of esophageal squamous cell cancer treated with neoadjuvant chemotherapy: unification of the AJCC staging system and the Japanese classification. Eur J Surg Oncol 2022;48:1760–67. 35094909 10.1016/j.ejso.2022.01.014

[15] Wong IYH Chung JCY Zhang RQ. A novel tumor staging system incorporating Tumor Regression Grade (TRG) With lymph node status (ypN-Category) results in better prognostication than ypTNM stage groups after neoadjuvant therapy for esophageal squamous cell carcinoma. Ann Surg 2022;276:784–91. 35876374 10.1097/SLA.0000000000005636

[16] Kang J Lee HP Kim HR. Validation of the post-neoadjuvant staging system of the American joint committee on cancer, 8th edition, in patients treated with neoadjuvant chemoradiotherapy followed by curative esophagectomy for localized esophageal squamous cell carcinoma. Surg Oncol 2020;35:491–97. 33130441 10.1016/j.suronc.2020.10.015

[17] Zhang Y Cao Y Zhang J. Lymph node ratio improves prediction of overall survival in esophageal cancer patients receiving neoadjuvant chemoradiotherapy: a national cancer database analysis. Ann Surg 2023;277:e1239–e1246. 35797545 10.1097/SLA.0000000000005450 PMC11225578

[18] The International ESODATA Contributors Group. Klevebro F Kuppusamy MK Low DE. ESODATA: benchmarking esophagectomy complications. Ann Esophagus 2020;3:36.

[19] Agha RA Mathew G Rashid R. Revised strengthening the reporting of cohort, cross-sectional and case-control studies in surgery (STROCSS) guideline: an update for the age of Artificial Intelligence. Premier J Sci 2025;10:100081.

[20] College of American Pathologists. Protocol for the examination of specimens from patients with carcinoma of the esophagus. Version 4.2.0.0. (Protocol posting date: June 2021). 2021. Accessed 6 September 2025. https://documents.cap.org/protocols/Esophagus_4.2.0.0.REL_CAPCP.pdf

[21] Mandard AM Dalibard F Mandard JC. Pathologic assessment of tumor regression after preoperative chemoradiotherapy of esophageal carcinoma. Clinicopathologic correlations. Cancer 1994;73:2680–86. 8194005 10.1002/1097-0142(19940601)73:11<2680::aid-cncr2820731105>3.0.co;2-c

[22] Strobl C Malley J Tutz G. An introduction to recursive partitioning: rationale, application, and characteristics of classification and regression trees, bagging, and random forests. Psychol Methods 2009;14:323–48. 19968396 10.1037/a0016973 PMC2927982

[23] Xie Y Luo X Li H. autoRPA: a web server for constructing cancer staging models by recursive partitioning analysis. Comput Struct Biotechnol J 2020;18:3361–67. 33294132 10.1016/j.csbj.2020.10.038 PMC7688999

[24] Pan-J-J Mai H-Q Ng WT. Ninth version of the AJCC and UICC nasopharyngeal cancer TNM staging classification. JAMA Oncol 2024;10:1627–35. 39388190 10.1001/jamaoncol.2024.4354 PMC11581663

[25] Du X-J Wang G-Y Zhu X-D. Refining the 8th edition TNM classification for EBV related nasopharyngeal carcinoma. Cancer Cell 2024;42:464–473.e3. 38242125 10.1016/j.ccell.2023.12.020

[26] Groome PA Schulze KM Mackillop WJ. A comparison of published head and neck stage groupings in carcinomas of the tonsillar region. Cancer 2001;92:1484–94. 11745226 10.1002/1097-0142(20010915)92:6<1484::aid-cncr1473>3.0.co;2-w

